# JAK/STAT signaling in diabetic kidney disease

**DOI:** 10.3389/fcell.2023.1233259

**Published:** 2023-08-11

**Authors:** Yingjun Liu, Wenkuan Wang, Jintao Zhang, Shuo Gao, Tingting Xu, Yonghui Yin

**Affiliations:** ^1^ Clinical Medicine Department, Shandong University of Traditional Chinese Medicine, Jinan, China; ^2^ Department of Endocrinology, Affiliated Hospital of Shandong University of Traditional Chinese Medicine, Jinan, China

**Keywords:** JAK/STAT signaling, diabetic kidney disease, etiology, treatment strategy, inhibitor

## Abstract

Diabetic kidney disease (DKD) is the most important microvascular complication of diabetes and the leading cause of end-stage renal disease (ESRD) worldwide. The Janus kinase/signal transducer and activator of the transcription (JAK/STAT) signaling pathway, which is out of balance in the context of DKD, acts through a range of metabolism-related cytokines and hormones. JAK/STAT is the primary signaling node in the progression of DKD. The latest research on JAK/STAT signaling helps determine the role of this pathway in the factors associated with DKD progression. These factors include the renin–angiotensin system (RAS), fibrosis, immunity, inflammation, aging, autophagy, and EMT. This review epitomizes the progress in understanding the complicated explanation of the etiologies of DKD and the role of the JAK/STAT pathway in the progression of DKD and discusses whether it can be a potential target for treating DKD. It further summarizes the JAK/STAT inhibitors, natural products, and other drugs that are promising for treating DKD and discusses how these inhibitors can alleviate DKD to explore possible potential drugs that will contribute to formulating effective treatment strategies for DKD in the near future.

## 1 Introduction

Diabetes mellitus (DM) is a chronic metabolic disease characterized by elevated blood sugar levels. According to the latest survey data of the International Diabetes Federation (IDF), there are approximately 537 million diabetics worldwide in 2021, which is projected to rise to 643 million by 2030 and 783 million by 2045 (Magliano and Boyko, 2021). Macrovascular complications [cardiovascular disease (CVD)] and microvascular complications [diabetic kidney disease (DKD), diabetic retinopathy (DR), and diabetic neuropathy (DPN)] are the main causes of death in diabetic patients (Melendez-Ramirez et al., 2010; Dal Canto et al., 2019). DKD, the main chronic complication of DM, occurs in approximately 40% of patients with DM. It seriously compromises the health, safety, and quality of life of patients and is the leading cause of end-stage renal disease (ESRD), chronic kidney disease morbidity, and mortality worldwide (GBD Chronic Kidney Disease Collaboration, 2020; Mohandes et al., 2023). Proteinuria, glomerulosclerosis, and tubulointerstitial fibers eventually develop as a result of excessive extracellular matrix (ECM) buildup, glomerular basement membrane (GBM) thickening, mesangial dilatation, glomerulus and renal tubule hypertrophy, and podocyte dysfunction in DKD (Kanwar et al., 2008). The factors that promote the onset and development of DKD are complex, including renin–angiotensin system (RAS) disorder, podocyte abnormality, fibrosis, immunity, inflammation, and aging. The treatment of DKD is currently limited to the control of hyperglycemia, hypertension, and other related risk factors. It nonetheless has the limitations of continuous disease progression, less selection of existing drugs, obvious drug side effects, and a single action target (Gnudi et al., 2016; Song et al., 2023). Therefore, it is critical to investigate more feasible approaches for its complex pathogenesis, including those dependent on the JAK/STAT pathway, which has been extensively studied recently.

The JAK/STAT pathway consists of ligand–receptor complexes, JAKs, and STATs. There are four members in the JAK family (JAK1, JAK2, JAK3, and TYK2) and seven members in the STAT family (STAT1, STAT2, STAT3, STAT4, STAT5a, STAT5b, and STAT6). Among the several subtypes, JAK2 and STAT3 have been the most frequently studied ones related to DKD (Brosius and Banes-Berceli, 2010; Chuang and He, 2010). The working principle of this pathway is that cytokines bind to their homologous receptors, leading to receptor dimerization and JAK phosphorylation. JAK-mediated phosphorylation recruits and phosphorylates STAT, and the phosphorylated STAT forms homo- or heterodimer, which is transferred to the nucleus, thereby controlling gene transcription and expression (Agashe et al., 2022). This axis is one of the core intracellular signaling pathways that regulate the signaling of a wide range of cytokines and growth factors, thereby mediating downstream events, such as inflammatory infiltration, immune response, repair, proliferation, differentiation, motility, and apoptosis in cells (Hu et al., 2023). The JAK/STAT pathway has received considerable attention, and its effects on regulating inflammation and immunity have been highlighted in clinical studies. Additionally, a variety of JAK inhibitors, such as baricitinib, ruxolitinib, and tofacitinib, have been approved and marketed for the treatment of rheumatoid arthritis, bone marrow fibrosis, ulcerative colitis, and other diseases (Villarino et al., 2017; Luo et al., 2021). JAK/STAT signaling is active and contributes to the development of renal injury in the DKD models, according to substantial evidence from animal studies, cell experiments, and renal biopsy research (Berthier et al., 2009; Huang et al., 2019). Related studies on the therapy of DKD with Jak inhibitors are ongoing. The *post hoc* findings from a phase 2 clinical trial (NCT01683409) showed that patients with DKD who were treated with baricitinib (JAK1 and JAK2 inhibitor) achieved noticeable improvements in lowering proteinuria and decreasing levels of inflammatory cytokines in urine and blood (Tuttle et al., 2018). In addition, another Jak inhibitor, ruxolitinib, was shown to reduce proteinuria and improve renal inflammation and fibrosis in diabetic rats (El-Kady et al., 2021). The activation of JAK/STAT signaling and the benefits brought about by Jak inhibitors may reflect the effects that the JAK/STAT pathway has on different factors related to DKD, and targeting this signaling is promising for the treatment of DKD.

Here, we have systematically combed out the critical functions of JAK/STAT signaling in the RAS system, immune response, inflammatory response, fibrosis, senescence, autophagy, and EMT, all of which are involved in related influencing factors of DKD. In addition, based on the important role of JAK/STAT signaling in DKD, we further summarize the potential drugs targeting the signaling, including JAK inhibitors, natural products, and traditional Chinese medicinal formulas, which would provide a promising direction for the effective targeted therapy of DKD.

## 2 The important role of the JAK/STAT pathway in DKD

### 2.1 Expression of JAK/STAT in DKD

**FIGURE 1:**
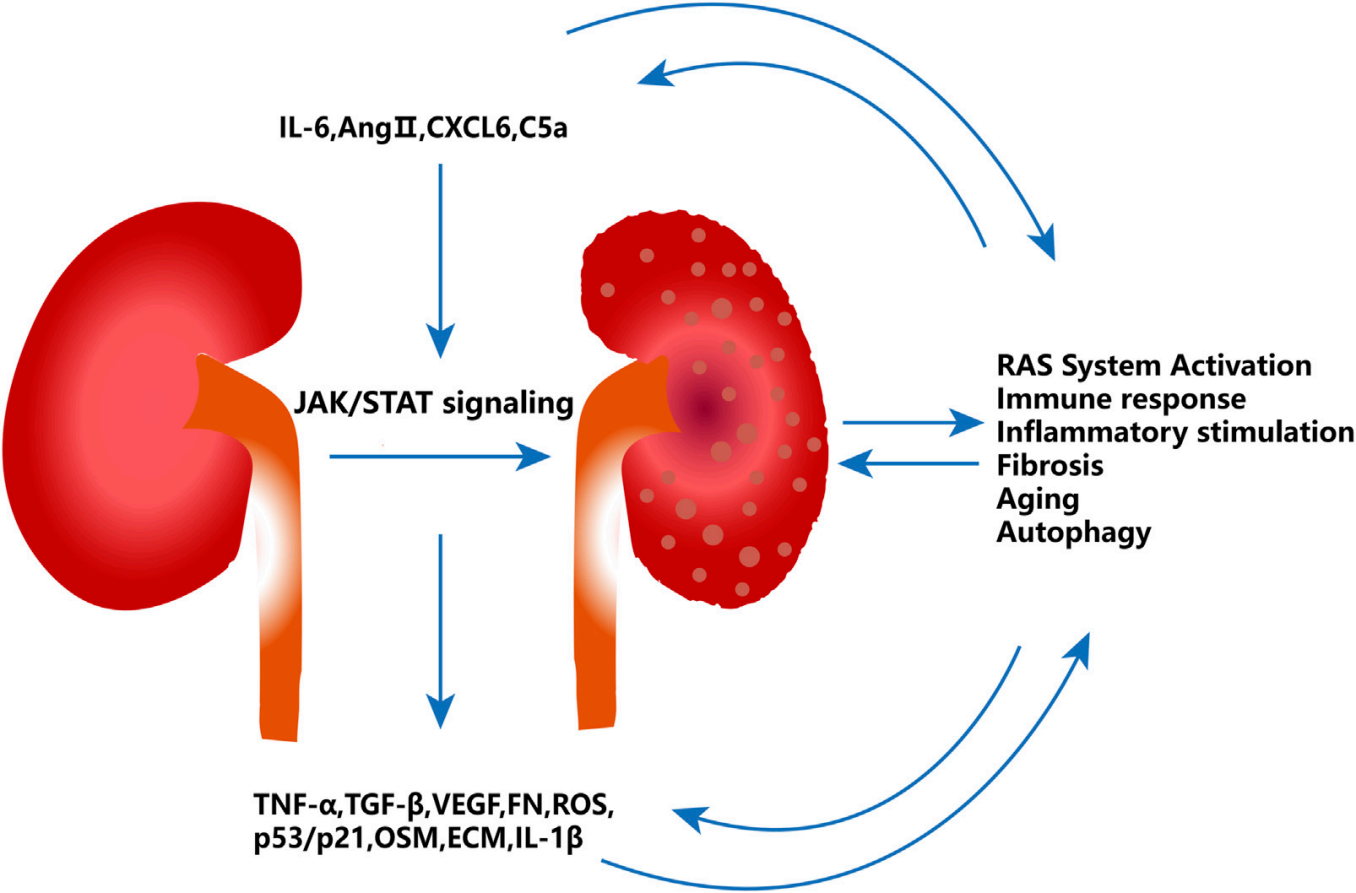
Role of JAK/STAT signaling in DKD. IL-6 and Ang II, which can be increased by CXCL6 and C5a, are the upstream factors that trigger JAK/STAT signaling. JAK/STAT activation further encourages the release of fibrosis and inflammatory factors, which in turn induce the immune response, inflammation, fibrosis, aging, autophagy, and EMT, and aggravates kidney damage symptoms such as glomerular sclerosis, renal interstitial fibrosis, and kidney volume loss.

JAK/STAT signaling is a significant mechanism pathway of metabolic diseases, including diabetes, obesity, and insulin resistance, and its activation is a crucial cause of renal injury induced by hyperglycemia (Gurzov et al., 2016). A number of elements, including the RAS system, immune defense, inflammatory response, fibrosis, aging, autophagy, and EMT, promote the DKD process via the JAK/STAT pathway (Figure 1). These elements also increase the expression of JAK and STAT in diabetic renal tissues (Marrero et al., 2006; Ricciardi and Gnudi, 2021). It has been reported that the glomeruli and tubulointerstitium of patients with DKD have significantly higher expression of JAK1-3, STAT1 and STAT3 mRNA, and protein, particularly JAK2, which activates JAK/STAT signaling and induces glomerular sclerosis and tubulointerstitial fibrosis (Berthier et al., 2009). Another intriguing finding is that JAK2 expression was significantly increased in the glomeruli of patients with early DKD, and JAK2 expression was more significantly upregulated in the tubulointerstitial region of patients with progressive DKD, which corresponds to the natural progression of pathological changes in glomerular damage followed by tubulointerstitial pathological changes in DKD (Berthier et al., 2009). In an experiment of transgenic mice with reduced STAT3 activity (STAT3SA/–mice) treated with streptozotocin (STZ), there was an increase in a few renal pathological changes, such as proteinuria, mesangial proliferation, and dilation; fibrotic factors; and immune and inflammatory cell infiltration, in the STAT3SA/– mice models (Lu et al., 2009). This study suggested that STAT3 may play a promoting role in renal pathological changes in diabetic mice. Studies have proved that the phosphorylation of STAT3 in renal tubular cells of type 1 diabetic C57BL/6 mice was enhanced, whereas the diabetic mice treated with STAT3 inhibitor S3I-201 resulted in the improvement of renal fibrosis, inflammatory response, and renal function (Zheng et al., 2019). The increased expression of JAK2 in podocytes and other glomerular and tubulointerstitial cells in Akita diabetic podocyte JAK2 mice further leads to DKD-related pathological changes, such as albuminuria, mesangial dilatation, glomerular sclerosis, and renal fibrosis (Zhang et al., 2017). Diabetic mice receiving JAK1 and JAK2 inhibitors showed that this treatment downregulates the expression of STAT3-dependent genes, such as Notch1, TGF-*β*, and CCR2, which are the driving factors of DKD (Zhang et al., 2017).

### 2.2 RAS

The RAS, which is distributed in the kidney, is an important endocrine system that regulates blood pressure and body fluid. The study and management of the RAS system are promising for the treatment of DKD (Malek et al., 2021). The RAS works through two pathways: the classical and alternative pathways (Panizo et al., 2021).

**FIGURE 2:**
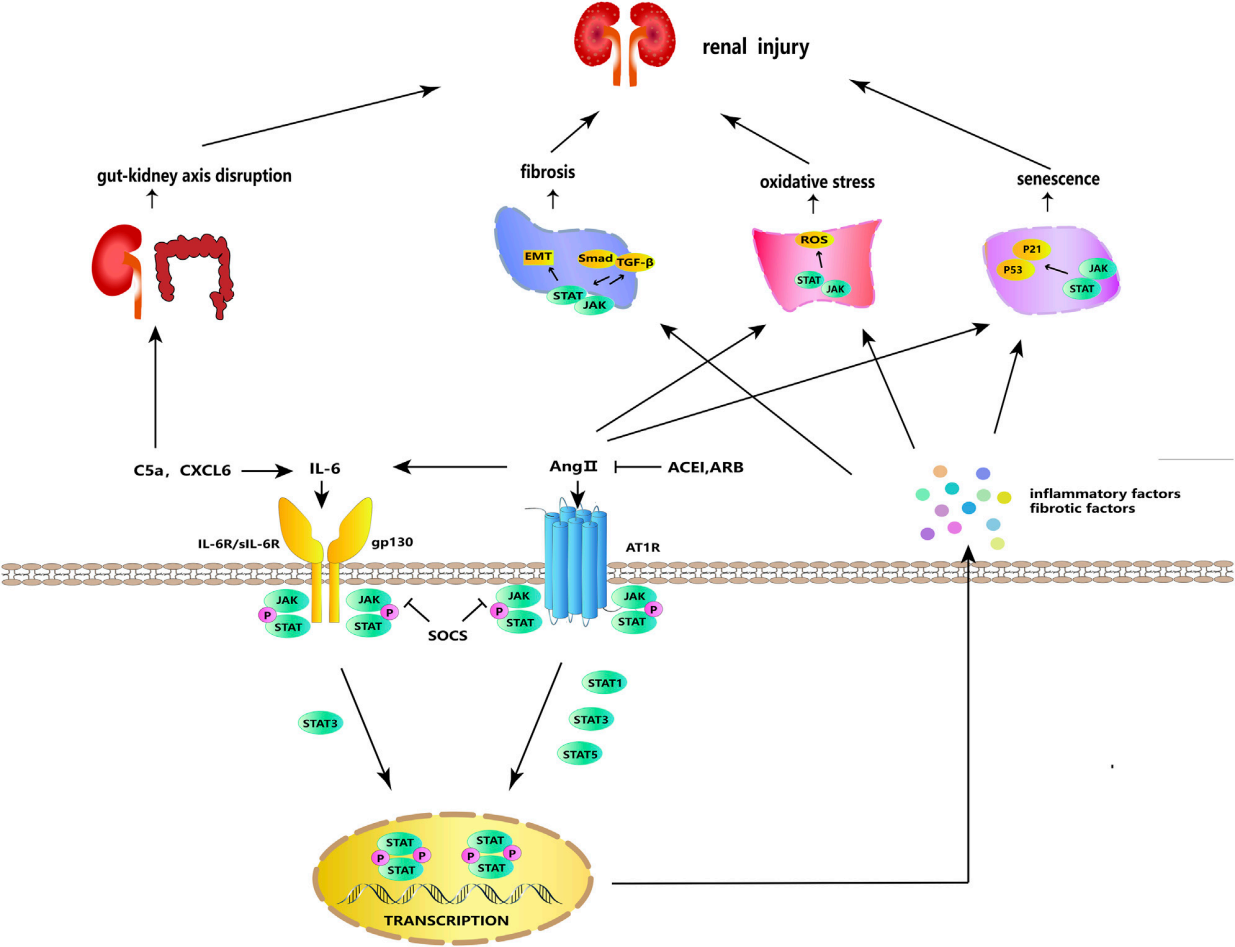
JAK/STAT signaling in cellular processes. IL-6 and Ang II can stimulate each other and bind to their respective receptors to activate JAK/STAT signaling. Activated JAK phosphorylates and dimerizes STAT, and the dimerized STAT is transferred to the nucleus to regulate the transcription and expression of genes, stimulating the further release of inflammatory and fibrosis factors. JAK/STAT signaling acts synergistically with ROS, TGF-*β*/Smad, and p53/p21 to participate in fibrosis, oxidative stress, aging, and other DKD-promoting incidents. SOCS, ARB, and ACEI have negative regulatory effects on this signaling. In addition, IL-6 can be activated by C5a and CXCL6, and C5a can participate in DKD via the gut–kidney axis.

In the classical pathway, renin secreted by juxtaglomerular cells promotes the conversion of angiotensinogen released by the liver into angiotensin I (Ang I), which is converted into angiotensin II (Ang II) under the mediation of angiotensin-converting enzyme (ACE) (Miller and Arnold, 2019). Subsequently, Ang II exerts its effects by attaching to angiotensin II receptors, including AT1R–AT4R, of which AT1R plays the major role (Mehta and Griendling, 2007). Ang II, a primary participant of the RAS, is actively expressed in local kidney tissues and promotes proteinuria, renal fibrosis, and abnormal apoptosis of podocytes under a high-glucose (HG) condition (Peng et al., 2014; Bahreini et al., 2021). It has been reported that STAT3, which may be activated by Ang II, boosts the synthesis and secretion of growth factors and cytokines (e.g., TGF-*β* and IL-6) in diabetic STAT3SA/–mice and further contributes to renal pathological changes (Lu et al., 2009). The activated IL-6, which can also be directly stimulated by Ang II, is also a stimulator of JAK/STAT and further stimulates the activation of JAK/STAT signaling (Moriyama et al., 1995). In a dose-dependent manner, Ang II raised the protein levels of *α*-SMA, fibronectin (FN), and collagen IV in cultured rat renal proximal tubular (NRK-52E) cells (Ni et al., 2014). In HG-induced glomerular mesangial cells (GMCs), Ang II promotes the phosphorylation of JAK2, STAT1, STAT3, and STAT5, as well as changes in collagen IV synthesis, cell growth, and proliferation, reflecting that Ang II may affect DKD kidney damage and fibrosis by activating the JAK/STAT pathway (Amiri et al., 2002). A key marker for the etiology of DKD is podocyte damage. Ang II has been found to activate the JAK2/STAT3 pathway, and this activation may be related to apoptosis, mitochondrial dysfunction, oxidative stress, and inflammatory response in mouse podocytes (Ji and Xu, 2016). Diabetic Sprague–Dawley (SD) rats given angiotensin II type 1 receptor blocker (ARB) and angiotensin-converting enzyme inhibitors (ACEIs) showed lower urine protein levels and less renal damage, and these improvements were linked to JAK/STAT suppression (Banes et al., 2004). Ang II acts through the G protein-coupled receptor AT1R and activates the JAK/STAT pathway by inducing JAK tyrosine phosphorylation, thereby affecting vascular smooth muscle (Marrero et al., 1998; Banes-Berceli et al., 2007). HG-induced increases in ACE and AT1 in NRK-52E cells were STAT3-dependent, and silencing STAT3 decreased the mRNA and protein levels of ACE and AT1 in renal tissues of diabetic C57BL/6 mice (Zheng et al., 2019). SHP1 is a redox-sensitive protein tyrosine phosphatase that inhibits Ang II- and AT1R-induced growth and inflammatory signaling by negatively regulating tyrosine kinase activation through dephosphorylation (Alvarez et al., 2008). SHP-2 has a high degree of homology and a comparable overall structure with SHP-1 (Zhang et al., 2000). SHP-1 inhibits the Ang II-induced JAK/STAT cascade by dephosphorylating JAK2, whereas SHP-2 enhances JAK2 phosphorylation and initiates the Ang II-induced JAK/STAT cascade (Marrero et al., 1998; Godeny et al., 2007). These findings revealed that, in the classical pathway, active Ang II and its receptor AT1RM could activate the JAK/STAT pathway and promote DKD progression (Figure 2).

ACE2 is a negative regulator of RAS, and in alternative pathways, ACE2 is a monocarboxypeptidase that converts Ang II into a hepta-peptide, Ang 1–7, which binds to the MAS G protein-coupled receptor (MAS-R) to negatively regulate the adverse effects mediated by Ang II and AT under the condition that pathological stimulation excessively activates RAS (Santos et al., 2018; Gheblawi et al., 2020). According to clinical research, the proximal tubules and glomeruli of patients with DM exhibit less ACE2 mRNA and protein, which has been shown to hasten the onset of renal injury (Reich et al., 2008). According to animal research, ACE2 deficiency alters the expression of the RAS gene in the kidneys of non-obese diabetic (NOD) mice and promotes podocyte apoptosis and renal fibrosis (Roca-Ho et al., 2020). Additionally, rhACE2 treatment improved renal dysfunction and oxidative stress injury in the kidneys of Akita mice by decreasing the expression of Ang Ⅱ in plasma and renal cortex, increasing the expression of ACE2 in the renal cortex, and increasing the expression of Ang 1–7 in plasma (Oudit et al., 2010). By suppressing the JAK2/STAT3 pathway, Ang 1–7 may alleviate the injury and inflammation of HG-induced human umbilical vein endothelial cells (HUVECs) (Chen et al., 2018). DKD causes the progression of diabetic microangiopathy. A previous study has shown that ACE2 can suppress apoptosis and inflammation by inhibiting the JAK2/STAT3 pathway, hence preventing the injury of HG-triggered human microvascular endothelial cells (HMECs) (Ren et al., 2022). The aforementioned findings suggest that in the alternative pathway, ACE2 inhibits the activation of the RAS and thus inhibits the JAK2/STAT3 pathway, reducing the inflammatory response and renal injury of DKD.

### 2.3 Immunity and inflammation

DKD is traditionally regarded as a non-immune-mediated disease. However, increasing evidence has shown that immune and inflammatory mechanisms play a closely related and crucial role in the onset and progression of DKD (Tesch, 2017). The activation of the immune system encourages an increase in the inflammatory response, resulting in the remodeling of renal interstitial structure and the development of renal fibrosis. The interaction of multiple immune pathways and inflammatory factors is an important mechanism for the development of DKD.

As a negative regulator of the JAK/STAT signaling pathway, SOCS proteins are important immune checkpoint molecules that regulate immune responses. They maintain self-tolerance and prevent autoimmunity and excessive immune inflammation by inhibiting the activation of T cells and negatively regulating the JAK/STAT signaling pathway (Palmer et al., 2015; Chikuma et al., 2017). Inflammation is a driving factor in DKD, and much evidence suggests that SOCS proteins are pro-inflammatory. SOCS2 can inhibit the activation of the JAK/STAT pathway and reduce the expression of MCP-1, TNF-*α*, and IL-6 inflammatory factors (Bao et al., 2015), and downregulation of SOCS expression can promote the polarization of macrophages to pro-inflammatory M1 type by promoting the activation of JAK/STAT signaling (Liang et al., 2017). Renal biopsies showed that the expression of SOCS proteins was elevated in the glomeruli and renal tubules of DM patients (Ortiz-Muñoz et al., 2010). In addition, HG induced high-expression SOCS protein to inhibit the phosphorylation of JAK 2/STAT1/STAT3 and STAT transcriptional activity in human mesangial cells (HMCs) and in human proximal tubular (HK-2) cells, proving that SOCS protein can negatively regulate the JAK/STAT pathway and lessen inflammation, fibrosis, and abnormal cell proliferation that are present in DKD (Ortiz-Muñoz et al., 2010).

The complement system is a part of the immune system. Many studies have shown that the complement system is tightly linked to the immune inflammatory microenvironment of DKD and that complement levels are elevated in DKD patients (Li et al., 2019; Duan et al., 2020). C5a, a component of the complement system, is an important inflammatory mediator that binds to the G protein-coupled receptor C5aR and activates its downstream cascade to initiate the inflammatory response (Guo and Ward, 2005). C5a can stimulate leukocyte infiltration into the damaged tissues and induce the release of pro-inflammatory factors, such as IL-1*β*, IL-6, and TNF-*α*, whereas the C5aR antagonist (C5aR) has the function of reducing DKD kidney tissue damage (Tan et al., 2022). According to a previous study, the renal tissues of DKD patients and type 2 diabetic db/db mice exhibited higher levels of C5a, which activated the STAT3 axis, thereby promoting renal inflammation and fibrosis and aggravating GEC damage, whereas C5aRA intervention reversed the aforementioned pathological changes (Li et al., 2021). Interestingly, C5a also promotes DKD progression by disrupting the gut–kidney axis. However, whether this disruption in DKD involves the JAK/STAT pathway needs further investigation (Li et al., 2021).

IL-6, an inflammatory factor with multiple effects, is produced by activated immune cells, including T cells, monocytes, macrophages, and neutrophils (Kang et al., 2020). It can act as an upstream inflammatory factor to activate the JAK/STAT pathway; produce TNF-*α*, IL-1*β*, and other pro-inflammatory factors; and promote M1 polarization to activate the inflammatory response (Fernando et al., 2014; Soni and Singh, 2021). To exert classical signaling effects, IL-6 is combined with the membrane-bound IL-6 receptor (mIL-6R), which is expressed only in certain types of cells. Moreover, to activate the trans-signaling, IL-6 can bind to the soluble IL-6 receptor (sIL-6R) (Feigerlová and Battaglia-Hsu, 2017). L6ST, also known as gp130, is the signaling subunit of the IL-6 receptor and binds to the IL-6/mIL-6R or IL-6/sIL-6R complex to activate the activation of intracellular signaling, including the JAK/STAT pathway and the SHP-2/JAK dependent Ras-Raf-MAPK pathway, thereby exerting downstream effects (Huang et al., 2021). Although classical signaling generally plays an anti-inflammatory role and trans-signaling plays an anti-inflammatory role, both classical signaling and trans-signaling promote podocyte injury in DKD (64). In addition to the paracrine effects, IL-6 can upregulate IL-6R and gp130 in podocytes in an autocrine manner, inducing podocyte dysfunction and injury in DKD (Jo et al., 2016; Lei et al., 2018). The IL-6 response is mediated by an IL6ST-STAT3-dependent mechanism (Huang et al., 2021). IL-6, sIL-6R, and sgp130 in the blood of DKD patients and membrane-bound IL-6 receptor (mIL-6R), sIL-6R, and gp130 in the renal cortex of diabetic C57BL/6 mice were upregulated, whereas the IL-6 signaling contributed to HG-induced podocytes injury through STAT3 phosphorylation (Lei et al., 2018). Furthermore, through interfering with the IL6T/STAT3 axis, miR-223-3p was reported to attenuate HG-induced damage in HUVECs and human renal glomerular endothelial cells (HRGECs) and may have a favorable effect in delaying DKD (Tang et al., 2023). These findings show that targeting the IL-6/JAK/STAT pathway to suppress the autocrine and paracrine effects of IL-6, as well as its classical signaling and trans-signaling, is the preferred therapy strategy for DKD (Figure 2).

In recent years, the role of chemokines in acute and chronic inflammation and fibrosis has been affirmed (Zhang et al., 2022). Chemokine CXCL6 is an important inflammatory factor. CXCL6 promoted HG-induced apoptosis and inflammatory response in HK-2 cells, according to a cell experiment. This promotion was related to CXCL6 upregulating the expression of p-JAK and p-STAT3 and activating the JAK/STAT pathway (Wang et al., 2021). In cultured rat renal interstitial fibroblasts (NRK-49F) cells, HG-induced CXCL6 overexpression increased cell proliferation and inflammatory cytokine production (e.g., IL-6 and TNF-*α*), whereas the JAK inhibitor AG490 reversed these effects (Sun et al., 2019).

In the inflammatory response, oxidative stress acts as a trigger and a regulator. In the state of continuous hyperglycemia, excessive production of reactive oxygen species (ROS) induces oxidative stress, causing changes in lipids, proteins, carbohydrates, and nucleic acids and contributing to the progression of DKD (Tanase et al., 2022). Jak-2 expression was upregulated in renal tissues of DKD patients and induced ROS production in mesangial cells (Berthier et al., 2009). Another intriguing study showed that ROS could link Ang II and AT1R with JAK protein in rat aortic smooth muscle (RASM) cells, thus activating the JAK/STAT pathway to play a key role (Schieffer et al., 2000). This linking effect might not be limited to available classical protein-binding motifs or related to O_2_^−^ anions produced by NAD(P)H in ROS-generating systems (Schieffer et al., 2000).

Tissue-resident memory T cells (TRM), a recently discovered population of non-circulating memory T cells, are mainly located in peripheral tissues (e.g., kidney, liver, skin, and genital organ) with limited circulation and play roles in some specific immune and inflammatory diseases (Park and Kupper, 2015). CD8^+^ TRM cells can be rapidly activated by antigens and produce a strong inflammatory response by secreting granzyme B (GZMB), TNF-*α*, and IFN-*γ* (Park and Kupper, 2015; Sun et al., 2019). The proportion of CD8^+^ TRM cells in DKD increases, which promotes podocyte injury and glomerulosclerosis (Li et al., 2022). Tofacitinib, a JAK/STAT pathway inhibitor, has been reported to effectively inhibit the production of TNF-*α*, IFN-*γ*, perforin, and GZMB by renal CD8^+^ TRM cells in MRL/lpr mice, thereby reducing proteinuria and inflammation and improving renal function by inhibiting CD8^+^ TRM cell function (Zhou et al., 2020). However, whether the effects of intervening in renal CD8^+^ TRM to improve renal inflammation and proteinuria by regulating the JAK/STAT pathway also occur in DKD needs further observation and research.

In conclusion, these findings suggest that immunity and inflammation are important participants in the progression of DKD and that JAK/STAT is a key signal for the role of immunity and inflammation in DKD.

### 2.4 Fibrosis

Renal fibrosis, a distinct pathological feature of DKD, is a crucial step in the progression from chronic kidney disease to renal failure (Zhang et al., 2021). The release of pro-fibrotic factors, such as TGF-*β*, PDGF, CTGF/CCN2, MMP2, and TIMP1, in response to HG stimulation stimulates the activation of fibroblasts and the formation of myofibroblasts, further stimulating myofibroblasts to secrete FN and collagen. Hence, the kidney undergoes ECM accumulation and interstitial fibrosis changes (Wang et al., 2017; Koszegi et al., 2019; Zhang et al., 2021). In addition, *α*-SMA is the most important marker protein expressed by myofibroblasts. It plays a vital part in the aforementioned process (Lucisano et al., 2013). HG can stimulate the expression of JAK2, STAT1, STAT3, and STAT5 in GMCs, whereas the inhibition of the JAK/STAT pathway can reduce the synthesis of TGF-*β* and fibronectin (Wang et al., 2002). The inhibition of STAT3 can attenuate the progression of renal fibrosis and reduce the production of macrophages and inflammatory cytokines (Tang et al., 2015). Furthermore, Zheng et al. (2019) found that STAT3 knockdown can improve renal function and reduce the expression of TGF-*β*1, VEGF, and other related fibrosis and collagen accumulation in C57BL/6 diabetic mice.

TGF-*β*, with multi-biological effects, is the central mediator of renal fibrosis and can induce the transcription of fibrosis factors in DKD through the Smad-dependent pathway (Chen et al., 2018). STAT3 impairs the formation of Smad3–Smad4 complexes and reduces Smad3 binding to chromatin, resulting in inhibition of Smad3-mediated transcriptional activation, and cross-talk between JAK/STAT and TGF-*β*/Smad promotes the fibrosis of DKD (Lan, 2012; Wang et al., 2016). Acetylation, such as phosphorylation, is also an essential protein modification that regulates gene transcription and expression. Smad3 acetylation and STAT3 acetylation are involved in the development of renal fibrosis (Li et al., 2010; Ni et al., 2014). Additionally, the inhibition of STAT3 acetylation may represent a new treatment for fibrosis-related DKD.

CXCL6, an important member of the ELR + CXC chemokine family, has pro-inflammatory and pro-fibrotic effects. CXCL6 was upregulated in kidney tissues and blood of DKD humans and rats, and renal fibrosis and renal injury were obvious in the kidneys of these models (Sun et al., 2019). Through the JAK/STAT pathway, CXCL6 may stimulate the production of TGF-*β*1, collagen I, collage III, MMP2, and MMP9, thereby speeding the renal fibrosis process in DKD SD rats (Sun et al., 2019).

PEDF is a secreted protein with multiple biological activities, such as anti-inflammation and anti-oxidation, and its anti-fibrosis effect is prominent in DKD (Wang et al., 2006; Hui et al., 2014). A cross-sectional study showed significantly elevated urinary PEDF in diabetic individuals, and an animal investigation found higher PEDF in the kidney and blood samples of diabetic SD rats (Chen et al., 2010). PEDF can inhibit the HG-induced activation of JAK2 and STAT1 in HMCs and reduce the expression of TGF-*β*2 and FN (Mao et al., 2013).

MiRNAs, which have been recently identified as DKD biomarkers, are a class of non-coding single-stranded RNA molecules that can participate in post-transcriptional gene expression regulation and thus many cellular processes (Piwkowska et al., 2022). One study showed that miR-150 antagonist induced an increase in markers of renal fibrosis (e.g., *α*-SMA, FN, and COL-1) in ICR mice, and this inducement was associated with the regulation of the SOCS1/JAK/STAT pathway, which is downstream of miR-150 (Luan et al., 2020). miR-196b-5p increased in renal tissues and serum of patients with DKD. Further experiments showed that miR-196b-5p in extracellular vesicles (EVs) promoted fibroblast proliferation and upregulated the levels of FN, col1A1, and *α*-SMA fibrosis-related factors (Hu et al., 2020). These modifications could be linked to the regulation of STAT3 and SOCS2 expression (Hu et al., 2020).

The aforementioned findings suggest that many factors can cross-talk with JAK/STAT signaling. Once activated, this signaling can promote the expression of pro-fibrosis factors and collagen, thus affecting the pathological changes associated with DKD fibrosis.

### 2.5 Cellular mechanisms: senescence, autophagy, and EMT

Renal cell senescence is considered a pathological feature of DKD and is affected by factors such as oxidative stress, inflammation, and accumulation of advanced glycation end products (AGEs) (Guo et al., 2020; Xq et al., 2021). Cell senescence, a biological program that plays a significant role in renal aging, has physiological and pathological implications. Its primary features include cell cycle arrest and senescence-associated secretory phenotype (SASP) (Docherty et al., 2019; Kumari and Jat, 2021). p53/p21, an important cause of cell cycle arrest in senescence, can be activated in response to telomere depletion and oxidative stress (Gambino et al., 2013; Anderson et al., 2019; Barnes et al., 2019). According to a survey, the knockdown of STAT1 by STAT1-siRNA prevented Ang II and H_2_O_2_ from inducing HMC senescence and reduced the levels of p53 and p21^Cip^ (Jiao et al., 2012a). The reduced p53 expression may have been caused by the STAT1-Mdm2-p53 pathway, and the decreased p21^Cip1^ may have been caused by both p53-dependent and p53-independent pathways (Jiao et al., 2012a; Engeland, 2022). In addition, Ang II receptor blockers were able to slow renal aging by inhibiting the JAK/STAT pathway (Zhou et al., 2010; Jiao et al., 2012b). Senescence-associated secretory phenotypes (SASPs), including IL-1*β*, IL-6, IL-8, and TGF-*β*, are secreted by senescent renal cells and directly affect the surrounding cells and further affect tissue function in an autocrine and paracrine fashion (Tan et al., 2022). IL-6 and TGF-*β*1 are upstream stimulating factors of the JAK/STAT pathway. Therefore, the production of SASPs in senescent cells may exacerbate DKD pathology by activating JAK/STAT signaling. Based on these findings, the inhibition of JAK and STAT is believed to delay renal aging, which may help identify potential targets for DKD anti-aging therapy.

Autophagy is a lysosome-dependent intracellular degradation pathway that maintains cell homeostasis by removing aberrant proteins and senescent organelles, and the deficiency of podocyte autophagy is a significant contributing factor to renal injury in DKD (Zhang et al., 2022). STAT3 regulates autophagy in cytoplasm, nucleus, and mitochondria in a context-dependent manner (You et al., 2015). Studies have shown that after HG stimulation, podocyte autophagy increased in the short term. However, after long-term HG stimulation, autophagy was inhibited, exacerbating kidney injury (Lenoir et al., 2015; Zhang et al., 2023). Podocyte autophagy and injury were observed in kidney biopsies of DKD patients with massive proteinuria, and further experiments in autophagy-related 5 (atg5) knockout mice proved the causal relationship between massive proteinuria and podocyte injury caused by insufficient autophagy (Yasuda-Yamahara et al., 2015). High glucose activated JAK/STAT signaling in STZ-induced C57BL/6 mice’s renal tissues and immortalized murine podocyte MPC-5 cells, inhibited autophagy, aggravated renal injury, and increased podocyte apoptosis. At the same time, the JAK/STAT pathway inhibitor, ruxolitinib, reversed the aforementioned conditions (Chen et al., 2021). These demonstrate the role of this pathway in DKD. However, further research and studies are needed to fully elucidate this role.

The process of epithelial-to-mesenchymal transition (EMT), a crucial process of renal fibrosis in DKD, permits renal tubular epithelial cells to acquire a mesenchymal phenotype by the breakdown of epithelial cell–cell connections and promotes the formation of myofibroblasts that can produce extracellular matrix (ECM) (Hu et al., 2022). Mesenchymal cell biomarkers, such as *α*-SMA, are upregulated during the process of EMT, whereas epithelial cell biomarkers, such as E-cadherin, are downregulated (Kriz et al., 2011). EMT is activated by various events and is thought to be a significant mechanism in the progression of renal fibrosis in DKD (Li et al., 2019). AGEs are the heterogeneous molecules formed by the non-enzymatic glycation of proteins, lipids, or nucleic acids under the HG condition and are involved in fibrosis, oxidative stress, inflammation, and apoptosis in DKD (Pastino et al., 2017; Rabbani and Thornalley, 2018; Rungratanawanich et al., 2021). SOCS is a negative regulator of JAK/STAT signaling. It has been proved that AGE-BSA significantly lowered E-cadherin expression while increasing *α*-SMA expression in HK-2 cells to induce EMT, and SOCS3 reversed the aforementioned expression by inhibiting the JAK2/STAT3 axis (Yu et al., 2017). Renal delivery of SOCS1 and SOCS3 alleviated proteinuria, reduced OSM expression and ECM deposition, partially restored the expression of CK18 and *α*-SMA, and improved renal fibrosis in the kidneys of diabetic CD-1 mice (Liu et al., 2014). These improvements may be achieved by attenuating STAT activity (Liu et al., 2014). The aforementioned findings suggest that JAK/STAT may play an integral role in driving EMT in DKD and that SOCS, a negative regulator of this pathway, critically influences this process.

## 3 Potential drugs targeting JAK/STAT for DKD treatment

### 3.1 JAK/STAT inhibitors

To date, JAK inhibitors have shown effective efficacy in a wide range of diseases, especially inflammatory and autoimmune diseases (Banerjee et al., 2017). Tofacitinib, an inhibitor of JAK1 and JAK3, and baricitinib, an inhibitor of JAK1 and JAK2, have been approved for the treatment of rheumatoid arthritis (Taylor, 2019). Additionally, ruxolitinib, an inhibitor of JAK1 and JAK2, has been approved for the treatment of myelofibrosis (Plosker, 2015). JAK inhibitors have also achieved results in clinical trials of autoimmune and inflammatory diseases, such as Crohn’s disease, ankylosis, ulcerative colitis, psoriasis, atopic dermatitis, and systemic lupus erythematosus (Schwartz et al., 2017; Kim, 2020). Although current drugs targeting JAK/STAT for the treatment of DKD are still in their early stages, their therapeutic effects are impressive. These animal experiments and clinical trials could provide vital insights into the treatment of DKD and lead to the identification of further JAK/STAT inhibitors (Table 1).

**TABLE 1:** Potential JAK/STAT inhibitors for DKD treatment.

Agent	Experimental model	Phase of development and status	Target	Function	Common adverse event	Reference
Baricitinib	DKD patients	Phase II completed (NCT01683409)	JAK1 and JAK2 inhibitors	Inhibit inflammation and reduce albuminuria	Anemia	Brosius et al. (2016); Tuttle et al. (2018)
Ruxolitinib	STZ-induced Wistar rat model, HG-induced MPC-5 cell model, and TGF-*β*1-treated NRK-52E cell model	Pre-clinical	JAK1 and JAK2 inhibitor	Inhibit inflammation and fibrosis and modulate podocyte autophagy	Not described	Bai et al. (2020); Chen et al. (2021a); El-Kady et al. (2021)
Nifuroxazide	STZ-induced SD rat model and UUO rat model	Pre-clinical	JAK2/STAT pathway inhibition	Inhibit oxidative stress, inflammation, apoptosis, and fibrosis	Not described	Elsherbiny et al. (2018); Said et al. (2018); Liu et al. (2019); Hassan et al. (2021); Liu et al. (2021)
Sinomenine	STZ-induced SD rat model	Pre-clinical	JAK2/STAT3/SOCS1 pathway inhibition	Inhibit inflammation, fibrosis, and apoptosis	Skin lesions and gastrointestinal discomfort	Zhu et al. (2021)
Silymarin	STZ-induced SD rat model	Pre-clinical	JAK2/STAT3/SOCS1 and TGF-*β*/Smad signaling pathway inhibition	Inhibit oxidative stress, inflammation, and fibrosis	Gastrointestinal discomfort	Chen et al. (2021b)
Total glucosides of paeony	STZ-induced Wistar rat model	Pre-clinical	JAK2/STAT3 pathway inhibition	Anti-inflammation	Not described	Wang et al. (2012)
Paeoniflorin	STZ-induced C57BL/6J mouse model	Pre-clinical	JAK2/STAT3 pathway inhibition	Anti-inflammation	Not described	Li et al. (2018)
Isoliquiritigenin	HFD/STZ-induced SD rat model	Pre-clinical	JAK2/STAT3 pathway inhibition	Inhibit inflammation, fibrosis, and oxidative stress	Not described	Sun et al. (2021)
*Momordica charantia*	STZ-induced Wistar rat model	Pre-clinical	JAK2/STAT3,5/SOCS3,4 pathway inhibition	Anti-inflammation	Not described	Elekofehinti et al. (2021)
Danzhi Jiangtang capsule	AGE-induced GMC model and HFD/STZ-induced SD rat model	Pre-clinical	JAK/STAT pathway inhibition	Inhibit inflammation and oxidative stress	Not described	Sun et al. (2019c); Fang et al. (2020)
ErHuang formula	HFD/STZ-induced SD rat model and HG-induced NRK-49F cell model	Pre-clinical	CXCL6/JAK/STAT3T pathway inhibition	Anti-fibrosis and anti-inflammation	Not described	Shen et al. (2020)
Liraglutide	db/db mouse model	Pre-clinical	JAK/STAT/SIRT1 pathway inhibition	Anti-inflammation	Nausea	Einbinder et al. (2016); Jacobsen et al. (2016); Zitman-Gal et al. (2019)
Vitamin D	RAW264.7 cell model and HG-induced GMC model	Pre-clinical	JAK/STAT pathway inhibition	Inhibit fibrosis and oxidative stress	Digestive and psychiatric symptoms	Zhang et al. (2019); Yang et al. (2021)
Probucol	HG-induced HMC	Pre-clinical	JAK2/STAT pathway inhibition	Anti-inflammation	Gastrointestinal disturbance	Du et al. (2010)
Fluvastatin	HG-induced GMC model	Pre-clinical	JAK2/STAT pathway inhibition	Anti-fibrosis	Hepatic impairment	Shi et al. (2008)

DKD, diabetic kidney disease; STZ, streptozotocin; HG, high glucose; TGF-*β*, transforming growth factor-beta; HFD, high-fat diet; GMC, glomerular mesangial cell; AGEs, advanced glycation end products; HMC, human mesangial cell; JAK, Janus kinase; STAT, signal transducer and activator of transcription; SOCS, suppressor of cytokine signaling; TGF-*β*, transforming growth factor-beta; CXC, (C–X–C motif) ligand; SIRT1, silent information regulator sirtuin 1.

#### 3.1.1 Baricitinib

Baricitinib, an oral small-molecule JAK inhibitor, with immunosuppressive effects, has been well developed in clinical studies for the treatment of rheumatoid arthritis (Fleischmann et al., 2017), atopic dermatitis (Simpson et al., 2021), and alopecia areata (King et al., 2022). A phase II, multicenter, double-blind, randomized, controlled clinical trial of type 2 diabetic patients with DKD, treated with the JAK1 and JAK2 inhibitor, baricitinib, found that subjects who received baricitinib for 24 weeks had significantly reduced albuminuria and up to 40% reduction in urine ACR compared with placebo subjects. In addition, the patient’s urine CXCL10 and CCL2 and plasma sTNFR1, sTNFR2, SAA, ICAM1, and VCAM1 levels were significantly reduced (NCT01683409). In this trial, the most commonly reported adverse event of baricitinib was anemia, which was defined by a reduction in hemoglobin and occurred mainly in patients receiving the higher dose, with modest rates of other adverse events (Brosius et al., 2016; Tuttle et al., 2018). A long-term safety study of baricitinib also reported its safety profile, which is consistent with the results of the aforementioned clinical trials (Taylor et al., 2022). However, existing findings indicate that the treatment time of baricitinib for autoimmune diseases is substantially shorter than that for DKD, and longer treatment may result in more serious anemia, infection, and other safety issues, a matter that needs further exploration and research. In addition, further large-scale clinical trials are required to determine the efficacy and safety of baricitinib in the treatment of DKD.

#### 3.1.2 Ruxolitinib

Ruxolitinib is a JAK1 and JAK2 inhibitor with 82.5% structural similarity to baricitinib (Schwartz et al., 2017). An animal study showed that ruxolitinib and ACEI enalapril had similar effects on reducing proteinuria in DKD Wistar rats. It significantly reduced the levels of serum cystatin C, TNF-*α*, NF-*κ*B, TGF-*β*1, vimentin, and other inflammatory and fibrosis markers, which have protective effects on DKD (El-Kady et al., 2021). Furthermore, in immortalized murine podocyte (MPC-5) cells, ruxolitinib reversed the situation that HG induced decreased expression of atg5, atg12, beclin1, and LC3 and increased expression of p62 and modulated autophagy disturbance (Chen et al., 2021). Ruxolitinib inhibited the expression of fibrosis factors such as α-SMA, collagen I, and Fn in TGF-*β*1-treated NRK-49F cells, as well as the expression of EMT transcription factors such as Snail and Twist in TGF-*β*1-treated NRK-52E cells (Bai et al., 2020). It suggested that ruxolitinib may reduce ECM, EMT, and renal fibrosis by downregulating TGF-*β*1. To date, there has been no clinical study of ruxolitinib in the treatment of DKD, and animal-relevant experiments have not been fully performed.

#### 3.1.3 Nifuroxazide

Nifuroxazide, a broad-spectrum antibiotic used to treat diarrhea, was found to inhibit STAT3 phosphorylation. It has been widely studied for its potential involvement in cancer treatment (Nelson et al., 2008; Bailly, 2019). The inhibition of STAT3 by nifuroxazide is achieved by inhibiting its upstream JAK2 and Tyk2. Nifuroxazide is now used as a potent inhibitor of the JAK2/STAT pathway. It shows the protective effects of DKD against renal injury, oxidative stress, inflammation, and fibrosis by inhibiting STAT3 in diabetic SD rats (Elsherbiny et al., 2018; Said et al., 2018). Nifuroxazide has also been reported to reduce oxidative stress and inflammatory responses by inhibiting the NF-κB/JAK2/STAT pathway, thereby relieving renal fibrosis in unilateral ureteral obstruction (UUO) rats (Hassan et al., 2021). In addition, it can improve glucose metabolism disorder, inhibit gluconeogenesis, relieve HG-stimulated insulin secretion disorder, and reduce oxidative stress and apoptosis (Liu et al., 2019; Liu et al., 2021). At present, there is no clinical trial report on nifuroxazide for the treatment of DKD. However, due to its long-term clinical application and high oral safety, it may be clinically tested more easily, showing a promising prospect for the treatment of DKD.

### 3.2 Natural products

Natural products, as a research hotspot for the treatment of various diseases in recent years, have the advantages of a diverse range of species, high safety, and wide pharmacological effects, which can compensate for the drawbacks of many existing medications, such as general efficacy and large side effects (Ginwala et al., 2019; Duarte et al., 2022). With the development of metabolomics, genomics, bioinformatics, and other technologies, it is possible to better develop and apply natural bioactive components to clinical practice (Atanasov et al., 2021). In addition, phytotherapy and traditional Chinese medicinal formulas have attracted great interest because they can exert the synergistic therapeutic effect of various natural products (Schmidt et al., 2008; Tang et al., 2021). Many studies have shown that the bioactive effects of natural products have great therapeutic potential for DKD. Based on these studies, it is of substantial significance to further explore the pathogenesis and treatments of DKD.

#### 3.2.1 Sinomenine

Sinomenine is extracted from *Sinomenium acutum* and has prominent anti-inflammatory and immunosuppressive effects (Lai et al., 2022). The drug delivery technique of sinomenine has been continuously improved in recent years, making it a viable drug possibility (Chen et al., 2022). Sinomenine has been shown to inhibit the expression of fibrosis-related proteins and inflammation-related factors such as IL-6 and ICAM-1 in renal tissue of SD rats with STZ-induced DKD and reduce renal cell apoptosis and renal fibrosis, which may be related to the regulation of the JAK2/STAT3/SOCS1 pathway (Zhu et al., 2021). Skin lesions and gastrointestinal discomfort have been identified as common side effects of sinomenine. These side effects are related to the stimulation of histamine release (Xiang et al., 2023).

#### 3.2.2 Silymarin

Silymarin, a flavonoid derived from the seed shell of silymarin, has the effects of anti-inflammation, anti-oxidation, anti-fibrosis, and promoting cell regeneration. It has been widely used as a liver protective agent in clinical practice (Aghemo et al., 2022). Silymarin exerts multiple effects of improving podocyte injury, renal fibrosis, oxidative stress, and inflammation level in the treatment of diabetic SD rats. These improvements are connected to the inhibition of JAK2/STAT3/SOCS1 and TGF-*β*/Smad signaling pathways (Chen et al., 2021). Clinical trials have shown that silymarin is safe and well tolerated at therapeutic doses, and adverse events (AEs) are mild, mainly gastrointestinal discomfort (Soleimani et al., 2019). However, teratogenicity has been observed in animal experiments (Soleimani et al., 2019). Despite the absence of relevant clinical trial findings, the evidence provided by animal studies suggests that silymarin should be taken with caution in pregnant women (Gholami et al., 2016; Gholami et al., 2017).

#### 3.2.3 Total glucosides of paeony and paeoniflorin

Total glucosides of paeony (TGP), which have remarkable anti-inflammatory and immune-regulating effects, are extracted from the root of *Paeonia lactiflora Pallas* (Zhang and Wei, 2020). TGP suppresses the activation of the JAK2/STAT3 pathway in the kidneys of diabetic Wistar rats, consequently inhibiting the infiltration, proliferation, and activation of macrophages, thus slowing the progression of DKD (Wang et al., 2012). Paeoniflorin, the active component of TGP, is also the main bioactive active component of TGP (Zhang and Wei, 2020). It can alleviate the infiltration of macrophages and inflammatory factors in the kidneys of STZ-induced C57BL/6J mice to achieve the purpose of protecting the kidney. This protection is related to the inhibition of the JAK2/STAT3 signaling pathway (Li et al., 2018). Both TGP and paeoniflorin are safe, with no noticeable adverse reactions found in clinical trials. Additionally, studies have shown that TGP combination with other agents can achieve better efficacy on the basis of reducing side effects (Jiang et al., 2020; Wang et al., 2022).

#### 3.2.4 Isoliquiritigenin

Isoliquiritigenin, a flavonoid extracted from glycyrrhiza, has multiple biological activities such as anti-inflammation, anti-oxidation, anti-tumor, and anti-diabetic complications (Peng et al., 2015). The therapeutic effect of isoliquiritigenin is to reduce IL-6 and ICAM-1, which are inflammatory factors, by inhibiting the JAK2/STAT3 pathway, relieving oxidative stress and renal fibrosis, and alleviating acute kidney injury in high-fat diet (HFD)/STZ-induced DKD SD rats (Sun et al., 2021). Furthermore, isoliquiritigenin usage has been associated with very few treatment-related AEs.

#### 3.2.5 Momordica charantia

*Momordica charantia* is a Cucurbitaceae plant containing proteins, saponins, flavonoids, and other substances. It has multiple biological effects, such as anti-diabetic, anti-tumor, and antibacterial properties (Jia et al., 2017). The bitter melon nanoparticles are prepared from *Momordica charantia* and can interfere with multiple inflammatory pathways. It was reported that the bitter melon nanoparticles alleviated the renal inflammatory response in diabetic Wistar rats by downregulating the expression of JAK2, STAT3, and STAT5 and upregulating the expression of SOCS3 and SOCS4 (Elekofehinti et al., 2021). Notably, the application market of nanomedicine has been expanding in recent years, and the safety and efficacy of nanomedicine have been widely recognized (Chen et al., 2022).

#### 3.2.6 Danzhi Jiangtang capsule

Danzhi Jiangtang capsule (DJC) is composed of six plant medicinal materials (Radix Pseudostellariae, Radix Rehmanniae, Rhizoma Alismatis, Cortex Moutan, Semen Cuscutae Chinensis, and Leech) and has been used in clinical practice to treat diabetes (Fang et al., 2020). *In vitro* studies demonstrated that DJC inhibited the upregulation of iNOS and COX2 in AGE-induced GMCs and reduced oxidative stress and the levels of TNF-*α*, IL-6, and MCP-1 inflammatory cytokines. These changes were associated with the inhibition of the JAK/STAT pathway (Sun et al., 2017). Meanwhile, an *in vivo* study showed that the DJC alleviated renal dysfunction and inflammatory injury in HFD/STZ-induced DKD SD rats, and these improvements were also associated with the inhibition of the JAK/STAT pathway (Sun et al., 2019).

#### 3.2.7 ErHuang formula

Furthermore, the ErHuang formula (EHF) is a traditional Chinese medicinal formula consisting of Astragali Radix, Rhei Radix et Rhizoma, Trigonellae semen, Vaccariae semen, Achyranthis bidentatae radix, Smilacis glabrae rhizoma, and Curcumae rhizoma (Shen et al., 2020). In HFD/STZ-induced DKD SD rats and HG-induced NRK-49F cells, EHF suppressed the expression of inflammation and fibrosis cytokines, such as TNF-*α*, TGF-*β*1, IL-6, MMP2, MMP9, collagen I, and collagen III, and decreased the p-STAT3 levels (Shen et al., 2020). It has been shown to treat DKD rats through its anti-inflammatory, anti-proliferative, and anti-fibrotic effects (Shen et al., 2020). CXCL6 increases the development of renal fibrosis by activating JAK2/STAT signaling (Sun et al., 2019). In addition, EHF reduces CXCL6 overexpression in HG-induced NRK-49F cells, suggesting that its effect on improving fibrosis may be mediated by inhibiting the CXCL6/JAK/STAT3 signaling pathway (Shen et al., 2020).

### 3.3 Other medications

#### 3.3.1 Liraglutide

Liraglutide is a member of the glucagon-like peptide-1 (GLP-1) family, which has been widely utilized in the treatment of type 2 diabetes. GLP-1 improves glomerular filtration rate, reduces proteinuria, and has a strong protective effect on the kidneys of DKD patients (Kawanami and Takashi, 2020). Its mechanism is related to the improvement of oxidative stress, inflammation, and fibrosis (Winiarska et al., 2021). SIRT1, a member of the sirtuin family, can ameliorate DKD by relieving oxidative stress, mitochondrial dysfunction, and fibrosis (Qi et al., 2022). Liraglutide reduces the inflammatory response and vascular endothelial damage in db/db mice (Einbinder et al., 2016), which may be related to its ability to inhibit the JAK/STAT pathway and cause the downregulation of the regulatory gene *SIRT1* of the process (Zitman-Gal et al., 2019). The most frequently reported AE of liraglutide is nausea, which is related to its mechanism of action in delaying gastric emptying, and this side effect is dose-dependent and transient (Jacobsen et al., 2016).

#### 3.3.2 Vitamin D

Vitamin D (VD) deficiency is associated with DKD and has multiple effects, including lowering urine protein, fibrosis, and endothelial damage (Hu et al., 2019). A cross-sectional study showed that T1DM patients with VD deficiency had higher albuminuria levels (Felício et al., 2016). TREM-1, an immune receptor that can amplify the inflammatory response, is upregulated in the renal tissues of DKD patients and DKD rats (Zhang et al., 2019). In the mouse macrophage cell line (RAW264.7), active VD can prevent the transformation of macrophages to the M1 phenotype in the HG environment. This prevention is related to the inhibition of HG-induced phosphorylation of STAT1 to reduce TREM-1 expression (Zhang et al., 2019). VD inhibited the upregulation of TGF-*β* and fibronectin expression and reduced oxidative stress in HG-induced GMCs. The associated alleviation of glomerular mesangial cell injury was related to the inhibition of JAK/STAT signaling by VD (Yang et al., 2021). VD is a common clinical medication with a low incidence of adverse events, most of which are digestive and psychiatric symptoms, such as nausea, vomiting, and drowsiness, which generally occur with VD overdose.

#### 3.3.3 Lipid-lowering agents: probucol and fluvastatin

Probucol is a cholesterol-lowering drug with an antiproliferative effect. A study has found that probucol can inhibit the proliferation of HG-treated HMCs and reduce the levels of inflammatory factors, TGF-*β*1 and CTGF. These inhibitory actions were related to the inhibition of the JAK2/STAT pathway (Du et al., 2010). In addition, fluvastatin, another lipid-lowering drug, can inhibit the activation of JAK2 and STAT proteins in GMCs induced by HG. This suppression was potentially related to the inhibition of excessive production of TGF-*β*1 and FN (Shi et al., 2008). The most frequently reported AE of probucol is gastrointestinal disturbance, and the most commonly reported adverse event for fluvastatin is hepatic impairment.

## 4 Conclusion and future prospects

Owing to its crucial function in the immune system, the JAK/STAT pathway has attracted increasing attention in the fields of autoimmune disorders and cancers. Despite the complexity and incomplete understanding of DKD’s pathophysiology, the current data are adequate to suggest that JAK/STAT signaling is a viable target for DKD therapy.

In this review, we have gathered sufficient evidence to conclude that JAK/STAT plays a role in DKD development: isoforms of JAK and STAT, mainly JAK2/STAT3, were upregulated in DKD patients and animal models. The upregulation of various cytokines, chemokines, and growth factors activates JAK/STAT signaling, which is involved in several critical elements of DKD, such as RAS, fibrosis, immune response, inflammatory response, senescence, injury, and autophagy. However, most studies on the JAK/STAT pathway have focused on autoimmune diseases. Therefore, there is still a lack of sufficient evidence on how this pathway is involved in the progression of DKD. A growing number of medicines are being tested in both animal and human clinical trials. However, it can be difficult to extrapolate results from these animal studies to people because of the differences between the two species. Although relevant progress has been achieved in therapy research, the specific mechanism is not yet clear and needs further investigation, among which the study of kidney biopsy may be a possible solution.

In this review, we also explore JAK/STAT inhibitors, natural compounds, and other drugs that target the JAK/STAT pathway and have prospects for the treatment of DKD. Studies have shown the multifaceted role of the JAK/STAT pathway and the necessity of combined multi-drug and multi-mode treatment in DKD. To properly incorporate JAK/STAT targeted agents into multimodal therapy, in combination with other antifibrotic therapies, anti-inflammatory therapies, immunotherapy, and physical therapies, we need to uncover more predictive biomarkers than merely pathway hyperactivation. Given the varying drug susceptibility of individuals, it is also our study’s focus to understand more about the variations in individual DKD genomes to assist us in building therapeutic regimens for diverse genetic changes. With the continuous progress of modern medical technologies, such as network pharmacology, metabolomics, proteomics, immunomics, and genomics, DKD is expected to be well-controlled and improved.
